# Uniform comparison of several drugs which provide protection from noise induced hearing loss

**DOI:** 10.1186/1745-6673-5-26

**Published:** 2010-09-01

**Authors:** Sharon Tamir, Cahtia Adelman, Jeffrey M Weinberger, Haim Sohmer

**Affiliations:** 1 Department of Otolaryngology and Head and Neck Surgery, Shaare Zedek Medical Center, POB 3235, Jerusalem 91031, Israel; 2 Speech & Hearing Center, Hadassah University Medical Center, POB 12000, Jerusalem 91120, Israel; 3 Department of Otolaryngology and Head & Neck Surgery, Hadassah University Medical Center, POB 12000, Jerusalem 91120, Israel; 4 Department of Physiology; Institute for Medical Research - Israel-Canada, Hebrew University-Hadassah Medical School, POB 12272, Jerusalem 91120, Israel

## Abstract

**Background:**

The ability of drugs to reduce noise induced hearing loss (NIHL) has been evaluated in diverse experimental conditions (animal species, noise intensities, durations, assessment techniques, etc), making it difficult to assess their relative efficacy. The present study was designed to provide more uniform comparisons and to allow to a better understanding of the mechanism of the NIHL. Methods: The drugs studied included furosemide (loop diuretic) and the antioxidants N Acetyl-L-Cysteine, vitamins A, C, E with the vasodilator magnesium. Mice were exposed to a continuous broadband noise (113 dB SPL for 3.5 hours) and the NIHL was assessed in all animals before noise exposure and 1 week after with auditory nerve brainstem evoked responses (ABR) to broadband clicks and to 8 kHz tone bursts.

**Results:**

Each of the drugs alone and in combination led to similar reductions in NIHL.

**Conclusions:**

The loop diuretic furosemide, by reducing the magnitude of the endocochlear potential in scala media, probably depressed active vibrations of the outer hair cells and basilar membrane, resulting in reduction of free radical formation during the noise exposure. The antioxidants N Acetyl-L-Cysteine and vitamins A, C, E with the vasodilator magnesium presumably counteract the free radicals. Thus, the administration of the antioxidants to animals in which free radical formation had already been reduced by previous injection of furosemide did not have an additional protective effect on the NIHL.

## Background

Noise induced hearing loss (NIHL) leads to a decrease in quality of life, and therefore it has become a major concern for many researchers. In their experiments, they have tried to determine possible strategies for intervention, ranging from prevention of noise exposure to protection and treatment.

Many research groups have conducted animal experiments in order to assess the efficacy of various drugs in protecting the inner ear from NIHL or in its alleviation. These experiments were conducted in many diverse ways: *different species *(such as chinchillas [1], rats [2], guinea pigs [3], mice [4,5]), with *different types of noise *(continuous broad band [4,5], continuous octave band [1,3] or impulse [2]), a *wide range of noise exposure durations *(ranging from 40 minutes [6] to six hours [1]), and *exposure intensities *(for example from 105 dB SPL [1] to 128 dB SPL [7]), with NIHL *assessed at various time periods after the exposure *(for example 1 to 3 weeks [1] or 24 hrs to 4 weeks [2] after noise exposure) *using diverse assessment techniques *(for example auditory nerve-brainstem evoked responses (ABR) to broad band clicks [4,5], ABR to 4-40 kHz tone bursts [2], inferior colliculus evoked potentials to 1-8 kHz tone bursts [1], distortion product otoacoustic emissions [8] and histology of the cochlea [1-3]), and *drugs administered *(for example salicylic acid [4]], N-acetyl-l-cysteine [2], vitamins [A, C and E] with magnesium [3], furosemide [5], idebenone [6] and non steroidal anti-inflammatory agents [7]). Due to this diversity in experimental design, it has become increasingly difficult to assess the degree of protection from NIHL that each drug confers and their relative efficacy.

The present experiment was designed to overcome these obstacles by enabling a more uniform comparison of several of the drugs found to provide protection from NIHL, and also to gain insight into the mechanism of NIHL. The experimental animals (male Sabra albino mice), noise exposure type and duration (continuous broadband noise for 3.5 hours, which causes an intermediate degree of permanent threshold shift in these mice), auditory threshold assessment technique (ABR thresholds to broadband clicks and 8 kHz tone bursts in order to enable rapid screening of threshold in a large number of animals) and the times of threshold assessment (before the noise exposure and one week after) were the same for all drugs tested. The drugs administered included anti-oxidants which can counteract free radicals produced by metabolic activity during the noise exposure, a loop diuretic (furosemide) which depresses the endocochlear potential, and combinations of these agents. The substances administered in the experimental groups were: furosemide alone, N Acetyl-L-Cysteine (NAC) alone, both furosemide and NAC in the same animals, vitamins A, C, E and magnesium (ACE+Mg), both vitamins ACE+Mg and furosemide in the same animals. Each drug was administered according to a protocol based on published data, as specified in Methods, and after preliminary experiments to determine the most effective protocol. It has already been shown that the injection of these drugs at the doses used, do not produce a permanent hearing loss, i.e. they are not ototoxic [5,9,10].

Since some of the drugs were in saline solution and others in oil, the control (vehicle-solvent) group was injected with saline and oil at equal volumes. The degree of protection provided by each of the drugs was evaluated as the difference between the final threshold shift (PTS) in the solvent vehicle control group and that in each drug group.

The results of these experiments can lead to possible treatment strategies and, in addition, based on knowledge of the presumed mode of action of the different drugs, it was hoped that they could provide insight into the mechanism whereby exposure to noise causes NIHL.

## Materials and methods

### Animals

Male Sabra (albino) standard laboratory mice, obtained from Harlan Laboratory (Jerusalem, Israel), at an initial age of 6-7 weeks, with mean body weights of 39.7 g (range 33-46g), were used in the study. They had normal hearing, defined as ABR thresholds to broadband clicks of 65 dB peak equivalent (pe) SPL or better. This is similar to the ABR thresholds of fat sand rats (Psammomys obesus) and the behavioral thresholds of normal hearing humans to the same broadband clicks delivered by the same insert earphones [11]. There is no change in hearing (no aging) over the duration of the study in control animals [4], and this served as a non-noise exposed control for test-retest and for aging over the period of the study (about 10 days).

### Noise exposure

In all experiments, in all groups, awake mice were exposed to broadband noise at an intensity of 113 dB SPL for 3.5 hours. The intensity and spectrum of the noise were periodically evaluated with a Bruel & Kjaer precision integrating sound level meter (type 2218) with a third octave filter. The noise peaked at 2 kHz and was 14 dB down at 250 Hz and 15 dB down at 8 kHz. (The spectrum of the noise has been reported; see ]12]). The intensity and duration of the noise exposure were chosen in order to produce an intermediate degree of PTS (24.2 dB, assessed with broadband clicks; 26.3 dB with 8 kHz tone bursts) in noise exposed animals injected only with the solvent of the drugs- (so that any protection provided by the drugs could be assessed; i.e. not too small a PTS which could indicate a total protection; and not too large degree of PTS which could lead to a"ceiling effect"). Up to three cages (with a maximum of fifteen animals per cage) were exposed together to the noise from the loudspeaker suspended centrally above them. Animals from experimental and control groups were exposed to the noise at the same time in shared cages.

### Anesthesia

All ABR recordings were carried out in anesthetized animals (Avertine 11.25 mg/kg intraperitoneally - IP). Additional anesthesia was administered as required in order to maintain areflexia when necessary.

### Auditory Brainstem Response

This study was designed to enable the rapid screening of auditory threshold (by recording ABR to broadband clicks and 8 kHz tone bursts) in a large number of animals (mice). This was important for the successful conduction of the study, based on the tight time restrictions involving drug injection, followed after 30 minutes by the noise exposure of the animals. ABR was recorded in each mouse in response to alternating polarity broadband clicks and to alternating polarity 8 kHz tone bursts (Blackman ramp, with a rise/fall time of 0.5 msec and a plateau of 5 msec) presented to the left ear by an insert earphone, using a Biologic Navigator Pro evoked potential system (Bio-logic Systems Corp., Mundelein, Ill., USA). Recording subdermal needle electrodes were inserted in the skin at the vertex with reference to the chin, with ground in a hindlimb. The stimuli were presented at a rate of 20/s from a maximal intensity of 120 dB pe SPL to below threshold in 5 dB steps. The responses were filtered (band pass 300-3000 Hz), amplified, and 128-256 responses were averaged and displayed vertex positive up. Threshold was defined as the lowest stimulus intensity required in order to elicit repeatable components (usually the first wave) of ABR in at least two out of three recordings. All initial ABR recordings were performed one to three days prior to starting the drug treatment protocol and noise exposure. The post-noise ABR threshold was assessed 7-8 days after the noise exposure.

The experimental protocol was evaluated and approved by the Hebrew University Hadassah Medical School Animal Care and Use Committee.

### Drugs

The dose regimens for each drug were adapted from those suggested by the research groups who studied each drug. The experimental animals and the control animals received the same total number of injections (identical degree of restraint and stress) while awake, so that the saline and oil control group also served as a control for the possible protection from NIHL by both restraint and stress induced by injection of substances IP and subcutaneously [13]. This was achieved by complementing actual drug injections with solvent injections when necessary, in order to reach a similar number of injections in each animal.

### Experimental Groups

**Figure 1:**
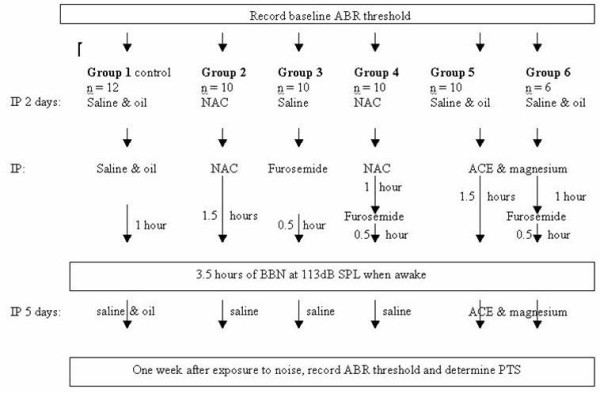
**Flow diagram of the experiment**. All of the animals received the same total number of injections, as drug injections necessary for the experiment were supplemented with control solution injections. For example, group #3 received a single injection of furosemide 0.5 hours before the exposure to 3.5 hours of noise, but in addition was injected with saline over the two preceding days and over the following five days in order to make up the number of injections. NAC - N-acetyl-l-cysteine; ACE - vitamins A, C and E; IP - intraperitoneal injection; PTS - permanent threshold shift; ABR - auditory nerve brainstem evoked response; BBN - broadband noise.

The experimental design of the entire study is outlined in the flow diagram (see figure 1).

Group I: Saline and oil solvent control

The saline and oil solvent control group (n = 12) received equal volumes of both saline (IP) and oil (subcutaneously) and these animals served as a control for all groups.

This control group received injections two days prior to noise exposure, a day prior to noise exposure and one hour prior to noise exposure. After noise exposure, these animals received daily injections for the following five consecutive days.

Group 2: N-acetyl-l-cysteine (NAC), an anti-oxidant, obtained from Sigma, Israel (n = 10). Based on preliminary experiments conducted in this laboratory and on reports in the literature [9], a treatment protocol for NAC was chosen. In those preliminary experiments, it was found that treatment with NAC twice daily prior to noise exposure, supplemented with an additional injection one and a half hours prior to noise exposure was similar to other protocols proposed in the literature [1] with respect to protection (i.e. reduction of NIHL). NAC was dissolved in 0.9% NaCl to the desired concentration of 325 mg/kg. The pH of the solution was adjusted to between 6.5-7.5 and was injected IP. A fresh solution was prepared each day.

These animals were injected IP twice daily with NAC, starting two days prior to noise exposure and continuing until the day of noise exposure, when the drug was injected one and a half hours prior to noise exposure. Saline was injected once daily after noise exposure for the following five consecutive days.

Group 3: Furosemide 100 mg/kg (n = 10) (a loop diuretic) was obtained from Teva Pharmaceutical Industries Ltd, Israel. The appropriate dosage, time of onset of the threshold elevation, its recovery and duration of threshold elevation plateau were based on a recent study in this laboratory [5]. These animals were injected IP twice daily with NaCl 0.9% on the two days prior to the noise exposure and, on the day of the noise exposure, half an hour prior to the noise exposure, these animals received a single injection of 100 mg/kg furosemide, which has been shown not to be ototoxic [5]. Saline was injected after noise exposure, once daily for the following five consecutive days.

Group 4: NAC and Furosemide (n = 10). These animals were injected IP twice daily with NAC 325 mg/kg two days prior to noise exposure, one day prior to noise exposure and one and a half hours prior to noise exposure. In addition, half an hour prior to noise exposure these animals received a single injection of furosemide 100 mg/kg. Saline was injected after noise exposure, once daily for the following five consecutive days.

Group 5: Vitamins A, C, E + Magnesium (n = 11). Based on published experiments [10], a treatment protocol was designed for this group. This group was injected with saline and oil two days prior to noise exposure and one day prior to noise exposure. All vitamins, as well as magnesium, were then injected one and a half hours prior to the noise exposure. Vitamins A and E were dissolved in vegetable oil while Magnesium and vitamin C were dissolved in NaCl 0.9%. The daily dose of vitamin A (Retinoic Acid, Sigma, Israel) was 20 mg/kg, of vitamin C (Ascorbic Acid, Sigma, Israel) was 200 mg/kg, of vitamin E ((+)-α-Tocopherol from vegetable oil, Sigma, Israel) was 65 mg/kg and magnesium (Mg Sulfate Anydrhous reagent Sigma, Israel) was given at a dose of 60 mg/kg. After the noise exposure, the injections were continued once daily for five consecutive days.

Group 6: Furosemide + Vitamins A, C, E + magnesium group (n = 6). This group was injected with saline and oil two days prior to noise exposure and one day prior to noise exposure. One and a half hours prior to noise exposure, each mouse in this group received the vitamins + magnesium and half an hour prior to noise exposure these animals were given a single injection of furosemide. Injections of both vitamins and magnesium were continued, once daily, for five consecutive days following noise exposure.

### Threshold shift

To assess the noise-induced threshold shift in the animals, ABR testing was performed in all animals (control and the various drug groups) at seven to eight days following noise exposure (at which time the threshold shift is permanent) using both alternating polarity broadband clicks and alternating polarity 8 kHz tone bursts. Post-exposure thresholds were subtracted from pre-exposure threshold measurements to calculate the degree of permanent threshold shift (PTS).

## Results

To assess noise-induced threshold shift in the animals, ABR testing was performed in all animals before and seven to eight days following the noise exposure. The initial mean ABR threshold in each group (see tables 1 and 2) was compared across all groups with one-way ANOVA. No significant difference was found between these initial thresholds (in response to click: F = 1.28; p = 0.28; in response to 8 kHz tone burst: F = 1.54; p = 0.19). In all groups, there was a significant difference (two-tailed paired t-tests with Bonferroni correction; p < 0.001) between the initial mean threshold of each group and the mean threshold obtained for that group one week after exposure to noise; that is, there was a significant PTS (the value of which was obtained by subtracting the initial mean threshold from the final mean ABR thresholds in each group -see tables 1 and 2) in each group when assessed with clicks and with 8 kHz tone bursts.

**Table 1:** ABR thresholds to broadband clicks.

Group	Initial threshold	Final threshold	PTS	P
Saline + oil (control) (n = 10)	58.75 ± 6.07	82.75 ± 7.52	24.16 ± 9.25	< 0.001

NAC (n = 10)	57.0 ± 6.74	73.5 ± 11.06	16.5 ± 8.51	< 0.001

Furosemide (n = 10)	61.0 ± 3.94	74.5 ± 7.61	13.5 ± 5.29*	< 0.001

Furosemide + NAC (n = 10)	59.0 ± 6.14	74.0 ± 8.75	15.0 ± 7.45*	< 0.001

Furosemide + ACE +Mg (n = 6)	58.33 ± 6.06	76.67 ± 7.53	18.33 ± 5.16	< 0.001

ACE + Mg (n = 7)	55.0 ± 5.47	69.54 ± 9.86	14.54 ± 9.34*	< 0.001

Mean ± SD of ABR thresholds to broadband clicks in dB pe SPL and the threshold shifts (PTS) in dB and the number of mice in each group tested. The results of two-tailed t-tests with Bonferroni correction comparing initial and final thresholds are shown (in the column of P). PTS in the groups marked * were significantly different from that in the control group - i.e. provided protection.

**Table 2:** ABR thresholds to 8 kHz tone bursts.

Group	Initial Threshold	Final Threshold	PTS	P
Saline + oil (control) (n = 10)	57.08 ± 8.64	83.33 ± 6.15	26.25 ± 9.56	< 0.001

NAC (n = 10)	55.0 ± 9.42	72.0 ± 10.32	17.0 ± 10.05*	< 0.001

Furosemide (n = 10)	62.0 ± 5.09	81.5 ± 7.09	19.5 ± 8.64*	< 0.001

Furosemide + NAC (n = 10)	55.5 ± 6.43	76.0 ± 7.37	20.5 ± 8.95*	< 0.001

Furosemide + ACE +Mg (n = 6)	58.33 ± 5.16	82.50 ± 6.89	24.17 ± 3.76	< 0.001

ACE + Mg (n = 7)	53.63 ± 8.09	76.81 ± 12.50	23.18 ± 12.30	< 0.001

Mean ± SD of ABR thresholds to 8 kHz tone bursts in dB pe SPL and the threshold shifts (PTS) in dB and the number of mice in each group tested. The results of two-tailed t-tests with Bonferroni correction comparing initial and final thresholds are shown (in the column of P). PTS in the groups marked * were significantly different from that in the control group - i.e. provided protection.

Results of one-way ANOVA comparing PTS of all groups were significant (click: F = 2.71; p < 0.05; 8 kHz tone burst: F = 3.60; p < 0.01) and therefore post hoc tests (Dunnett's test, SAS software package) were performed to determine which experimental groups were different from the control group (i.e. which drug provided significant protection). With respect to click stimuli, all experimental groups showed in general a lower mean PTS than the solvent control group, though not all were significant: a significant difference (p < 0.05) was found for the following groups: furosemide, furosemide and NAC and the vitamins + magnesium group. With respect to the NAC treated group and furosemide + vitamins + magnesium treated group, the PTS was smaller than that in the solvent control group, though the difference was not significant.

All experimental groups also showed a lower mean PTS than the control group in response to the 8 kHz tone burst stimuli, but not all were significant; a significant difference was found for the furosemide group, NAC group, and the furosemide and NAC group. Both the vitamins + magnesium group and the furosemide + vitamins + magnesium group did not show a significant difference when compared to the solvent control group.

## Discussion

Due to the difficulty in comparing the results of different drug treatments in alleviating NIHL, we decided to conduct the present study, based on a more uniform experimental protocol in order to assess the efficacy of the various drugs at dose regimens which are not ototoxic. The entire study was conducted on the same species of animals, with similar ages and weights, same spectrum and duration of the noise exposure, same total number of injections, and the same auditory threshold assessment protocol. The degree of PTS was determined by recording and comparing, in all animals, ABR threshold in response to broadband clicks and 8 kHz tone bursts, and the results reflect this assessment protocol, and likely represent a large extent of the cochlea, since broadband clicks deliver a wide range of frequencies. This study has accordingly shown that furosemide, NAC and vitamins A, C, and E with magnesium all provide"protection"; i.e. in each of these groups there is a smaller PTS than that in the vehicle (solvent) control group. Since in the control group, the number of injections was the same as that in the experimental groups, the results are not due to the protective effect (conditioning) of the restraint and injection of the animals [13]. Differences in the degree of effectiveness of these drugs in the present study, compared to those reported by others, each in different animal species, types, intensities and durations of noise, assessed at different times after the exposure, etc, may well be due to lack of uniformity between the studies.

Since a single injection of furosemide at an appropriate time (30 minutes) *before *the broadband noise exposure (but not after) was effective in providing protection, it is likely that the protective effect of furosemide is related to its reversible depression of the endocochlear potential [14], which is one of the main electrochemical gradients required for auditory transduction, and reduction in the magnitude of the endocochlear potential. This leads to depression of the cochlear amplifier [15], with smaller active displacements of the outer hair cells (OHCs) [16] within a short period after its injection. Thus, at the time of the noise exposure, active displacements were likely depressed, leading to the protection. This result with furosemide is similar to the protection from NIHL provided by a single injection of salicylic acid (the active component of aspirin) before the noise onset (but not after) [4]. Salicylic acid acts as a competitive antagonist of the motor protein prestin in the OHCs, reducing active displacements of OHCs. Salicylic acid therefore also reversibly depresses the cochlear amplifier (though by a different mechanism than that of furosemide) so that during the noise exposure the active OHC displacements are reduced. Therefore it seems that the protection provided by these two drugs is due to the reduced active displacements of the OHCs and basilar membrane, with lower metabolic demands at the time of the noise exposure.

The anti-oxidants administered in this study (NAC; vitamins A, C, and E with the vasodilator Mg) were also protective more or less to the same extent and similar to that of furosemide; in fact when using broadband click stimuli to assess ABR threshold, furosemide was the most effective (administration of furosemide to this group lead to the smallest PTS), while with 8 kHz tone burst stimuli, the most effective drug was NAC. The anti-oxidants serve to reduce harmful effects of the excessive release of free radicals (such as reactive oxygen species, ROS) which occurs during and after the noise exposure. Elevated levels of ROS are produced as part of the metabolic processes involved in maintaining adequate electro-chemical gradients (with greater metabolic demand) required to continue auditory transduction in the presence of the noise exposure. The elevated ROS can lead to metabolically initiated structural damage to sensitive cochlear structures [17].

The finding that the loop diuretic furosemide (in a single injection) and the anti-oxidants each provide more or less the same degree of protection, coupled with the result that the administration of both types of drugs to the same animals (the loop diuretic furosemide together with the anti-oxidant NAC, or furosemide together with vitamins A, C, and E with Mg) provides no additional protection over that provided by each drug alone, have implications for understanding the mechanism of the NIHL following continuous broadband noise exposure. One can suggest that following furosemide injection, the resulting depression of the cochlear amplifier leads to the synthesis of lower levels of ROS, with less metabolically induced structural damage; while the damage due to elevated levels of ROS is reduced by the anti-oxidants. Thus in the presence of furosemide, the levels of ROS produced are lower so that the addition of anti-oxidant does not provide additional protection over that provided by the furosemide (thus precluding a synergistic effect of the two drugs). These considerations support the suggestion that the NIHL following exposure to broadband noise is due to excessive release of ROS which disrupts sensitive elements in the cochlea. Furosemide, producing a smaller metabolic demand as a result of the depression of the cochlear amplifier, leads to the release of lower levels of ROS, with less damage, whereas anti-oxidants counteract the elevated ROS levels induced by the noise exposure.

It is interesting to point out that none of these drugs provided total protection (as reported by others as well [1,3]); i.e. there was still a residual PTS following exposure to the intensity and duration of the noise used in this study. It is not likely that this residual PTS is the result of administration of inadequate drug levels because injecting higher concentrations of furosemide does not produce greater depression of the endocochlear potential [18], and a greater number of NAC injections than used here was accompanied by less protection [2] and can lead to pulmonary toxicity [19]. Therefore it is possible that other factors (in addition to the elevated ROS levels) such as necrotic and/or apoptotic damage may be contributing to the NIHL.

The drug injections in this study were most effective if their administration began before the noise exposure (e.g. furosemide and the anti-oxidants NAC or vitamins A, C, and E with the vasodilator Mg) and continued after the exposure (anti-oxidants NAC or vitamins A, C, and E with Mg), as also reported by others [3].

However in the search for optimal therapeutic treatment options, it would be helpful to have drugs which could be delivered after an unexpected noise exposure. Also, furosemide is not a desired treatment option since it is a diuretic and can lead to electrolytic imbalance. The inclusion of furosemide in the present experimental study was intended to gain insight into the mechanism of the NIHL, and not as a feasible treatment option. Another drug evaluated in this laboratory, salicylic acid [4] is also not always desirable since it is an anti-coagulant and can cause excessive bleeding. Therefore it would be worthwhile in future study of drugs with potential to reduce NIHL, to assess drugs with other modes of action, for example anti-apoptosis [20] and other anti-inflammatory [7] agents, especially if they can serve to"rescue" the noise exposed ear from hearing loss. In the future, the effectiveness of all of these drugs in preventing NIHL from impulse noise (fire arms) should also be evaluated.

## Conclusions

The NIHL induced by exposure to broadband noise could be due mainly to the elevated metabolism required to maintain adequate electro-mechanical gradients needed for transduction. This leads to release of excessive free radicals which exceed the levels of intrinsic antioxidants in the tissue. Thus drugs such as furosemide (and salicylic acid) which reduce active cochlear mechanics lead to reduced metabolic demand, with lower levels of free radical production. The additional administration of antioxidants will then not be as effective as when the antioxidants are given alone.

## Competing interests

The authors declare that they have no competing interests.

## Authors' contributions

ST and CA contributed to the study equally: conducted the study and contributed to the writing and statistics. JMW contributed to the collection of the data. HS conceived of the study and participated in its design, coordination and drafting the manuscript. All authors read and approved the final manuscript.
